# Analysis of Signal Transmission Efficiency in Semiconductor Interconnect and Proposal of Enhanced Structures

**DOI:** 10.3390/mi15101207

**Published:** 2024-09-28

**Authors:** Tae Yeong Hong, Sarah Eunkyung Kim, Jong Kyung Park, Seul Ki Hong

**Affiliations:** Department of Semiconductor Engineering, Seoul National University of Science & Technology, Seoul 01811, Republic of Korea

**Keywords:** current transmission optimization, high-density electronic devices, area ratio, interconnect, reliability improvement, bonding structure

## Abstract

As the demand for high-density, high-performance technologies in semiconductor systems increases, efforts are being made to mitigate and optimize the issues of high current density and heat generation within interconnects to ensure reliability. While interconnects are the most fundamental pathways for transmitting current signals, there has been relatively little research conducted on them compared to individual unit devices from the perspective of overall system performance. However, as integration density increases, the amount of loss in interconnects also rises, necessitating research and development to minimize these losses. In this study, we propose a method to analyze power efficiency by utilizing the differences between simulation results and measured results of interconnect structures. We confirmed that the difference between theoretical resistance values and actual measured values varies with the contact area ratio between metal lines and vias, and we analyzed the power efficiency based on these differences. Using the findings, we proposed and validated a structure that can improve power efficiency. This study presents a method to analyze power efficiency and suggests ways to achieve higher power efficiency within the limited specifications of interconnects. This contributes to enhancing power efficiency and ensuring reliability, thereby preserving the performance of the overall system in highly integrated semiconductor systems.

## 1. Introduction

The rapid growth of advanced scientific technologies, such as on-device AI, autonomous driving, and high-performance computing, is increasing the demand for highly integrated and high-performance semiconductors. As the integration and performance of semiconductors continue to advance, various challenges are emerging in the field of semiconductor development [1,2,3]. This trend is causing saturation in semiconductor system development. Due to the Boltzmann limit, power efficiency has become a crucial issue in modern electronic devices operating at smaller scales. Additionally, in the post-Moore era, it is predicted that the number of transistors will continue to increase annually, leading to challenges in energy efficiency improvements due to downsizing physical limitations, semiconductor materials, heat management, and dissipation [4,5].

**Figure 1:**
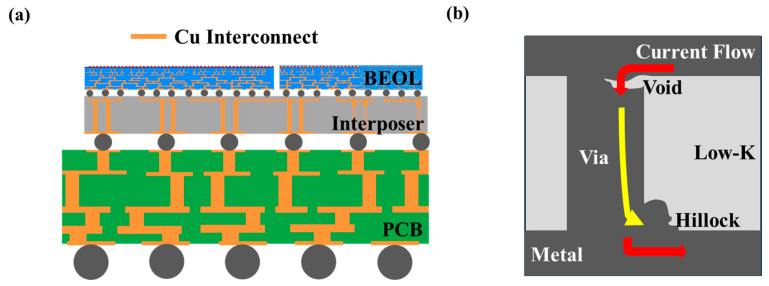
(**a**) Interconnect structure of BEOL, interposer and PCB on semiconductor system. (**b**) Interconnect representative defect: schematic of electro-migration.

New forms of field-effect transistors (FETs) are being researched to overcome these challenges [6]. However, even if excellent transistor prototypes are developed, their performance cannot be fully utilized if the interconnects do not function properly. Fundamentally, the Back-End-of-Line (BEOL) serves as the interconnection structure inside chips, which are densely integrated and feature compact paths. It connects each transistor to form circuits and acts as the starting point for signals coming out of the chip and the endpoint for external power. Additionally, it serves as the primary cooling channel, conducting heat generated in the chip encapsulated with epoxy to the outside, thereby lowering the internal temperature. As shown in Figure 1a, interconnection structures formed not only in BEOL but also inside the interposer and PCB consist of plugs called ‘metal lines’ and ‘vias’ [7]. These interconnect structures must effectively deliver external power to individual transistors to ensure the overall system reliability. However, the downsizing of the interconnects to sub-nanometer scales leads to increased current density due to resistance growth and heat generation, exacerbating electro-migration (EM). As shown in Figure 1b, EM phenomena can cause defects such as voids and hillocks, which, in severe cases, can lead to shorts. According to industry-standard IPC-9701A guidelines, the mean time to failure is 1065 h under a current of 1.4 A and at 125 °C. In 44 days and 9 h, a breakdown due to EM will occur somewhere in the interconnect. In this way, EM phenomena are one of the critical issues that negatively impact the yield and reliability of products [8,9,10]. To address this issue, continuous research is being conducted in materials engineering and electronic engineering fields for advanced interconnects. Research is actively underway on dielectric material development such as Fluorosilicate Glass (FSG), dense organosilicate glass (OSG) Low-k, Porous Low-k [11,12,13,14,15,16], and stable mechanical [17], thermal, chemical [18], and physical stability under processing conditions for integration with other materials, compatibility with other materials [19], crosstalk noise due to parasitic capacitance unintentionally formed during BEOL design [20], and reduction in power dissipation and RC Delay [21]. The following studies highlight the importance of improving interconnect reliability, indicating that significant efforts are required to secure overall performance and reliability.

We are currently conducting research to optimize the interconnect structures responsible for current transmission and distribution. To achieve this, we utilize Finite Element Method (FEM) analysis for modeling and analyzing the interconnects. In this study, we predicted the current density and resistance based on the intrinsic properties of the materials, depending on the shape of the interconnects. In a semiconductor system, the number of interconnects and metal bonding surfaces is exceedingly large. Due to their close proximity, interconnect capacitance and coupling capacitance exist, leading to significant interference in terms of signal efficiency and heat generation. However, this study focused on analyzing the independent impact of the interconnects rather than considering the entire system. We believe that analyzing at the system level would make it difficult to isolate and interpret the effects of each component due to the inclusion of various external variables, not just those of the interconnects themselves. Therefore, we prioritized understanding the impact of the interconnect components in isolation. By identifying the influence of these independent elements, we believe that it will also be useful in future system-level analyses when trying to disentangle the effects of individual components. We then compared these predictions with actual measurements to observe changes in resistance differences. By analyzing how the differences between theoretical values and actual measurements vary with the shape of the interconnects, we identified the configurations that result in additional current losses. Based on this analysis, we used FEM simulation to determine structures that can prevent these additional current losses and verified these findings through the fabrication of actual samples. The FEM analysis for this study was conducted using the Analysis Tools within Ansys Simulation (Workbench). The specific tools utilized were electric and steady-state Thermal, which were linked together and executed as separate projects. In this process, the Joule Heat values obtained from the electric analysis were used as input in the steady-state thermal analysis, along with the material properties, to convert the heat into temperature results. For the simulation model, Copper Alloy provided by Ansys Simulation was applied to both the metal lines and vias. The characteristics of this material are as follows: (Density: 8.3 × 10^−15^ kg/μm^3^, Isotropic Elastic structure, Young’s Modulus: 1.1 × 10^5^ MPa, Isotropic Thermal Conductivity: 4.01 × 10^8^ pW/μm·°C, Electric Isotropic Resistivity: 16.94 [Mohm × μm, 20 °C]). This methodology was employed to ensure accurate simulation results that reflect the real-world behavior of the materials and structures under study.

The analysis and the proposed improvement models can be utilized to enhance the design of interconnects, which has become increasingly important in the highly integrated semiconductor systems of today.

## 2. Experimental

**Figure 2:**
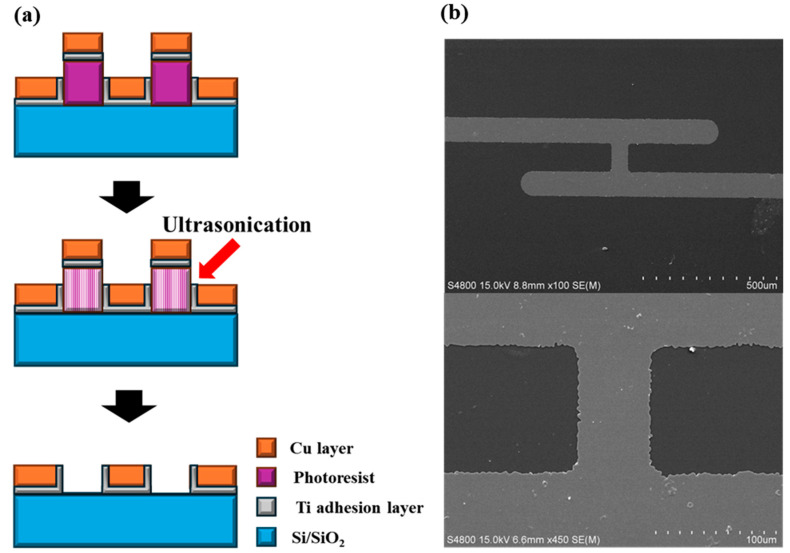
(**a**) A schematic diagram of the lift-off process and metal deposition for analysis sample fabrication. (**b**) SEM images of the implemented general sample model.

In this study, based on the current–voltage measurement results according to the interconnect structure, we compare the theoretical values and actual measured values through resistance conversion to determine the occurrence of additional power loss. For this measurement analysis, samples were fabricated and evaluated by simplifying a three-layer structure consisting of metal line/via/metal line into a flat 90-degree configuration. This approach eliminates the need for the CMP process and enhances experimental efficiency through a single deposition and a single lift-off process. Figure 2a outlines the sample fabrication process. The substrate used was a Si wafer with a 300 nm SiO_2_ layer. On this, photo patterning was performed, followed by the deposition of a 100 nm Cu layer (including a 10 nm Ti adhesion layer) using sputtering. The interconnect simulation samples were then fabricated through a lift-off process accompanied by ultra-sonication. Figure 2b shows the SEM images of the fabricated samples. The top and bottom parts simulate the metal lines, while the sections perpendicular to them simulate the vias. In this study, we maintained the specifications of the metal line while varying the size of the via. We then applied the same voltage and performed the analysis by using the difference between the theoretically calculated resistance values and the measured resistance values.

## 3. Study on the Mechanism for Sample Analysis Verification

The method we used for power loss analysis through resistance value comparison involved comparing theoretical values with actual measured values to observe any changes in the differences. The measurement samples are structured to simulate metal line/via/metal line, and we varied the size of the vias in these samples.

**Figure 3:**
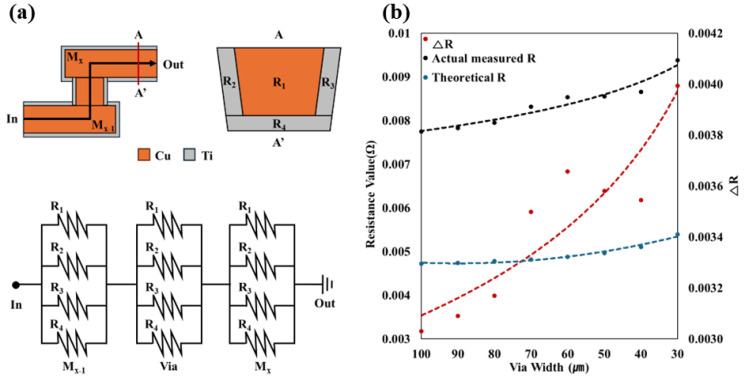
(**a**) The method for calculating theoretical resistance values. (**b**) A graph showing the theoretical values, actual measured values, and the differences between them according to via specifications.

The theoretical resistance values of the samples were calculated as shown in Figure 3a. Although formed using Cu, Ti is deposited as a barrier, resulting in a cross-section like that in Figure 3a. This means that Ti on three sides and Cu in the middle are connected in parallel. Additionally, since we simulated a metal line/via/metal line structure, three of these parallel structures are connected in series. Using the resistivity of Ti and Cu, and the length values measured with an optical microscope after fabricating the simulated samples, we calculated the theoretical resistance values. We then applied voltage to the pattern, measured the current, and used V = IR to determine the actual resistance value, comparing it with the theoretical resistance for each pattern. The theoretical and actual values inherently include resistance elements from measurement equipment (probe station, tips, etc.) and measurement errors. However, if these were the only factors, the difference between theoretical and actual values would remain relatively constant regardless of pattern changes, since these are fixed resistance values. If there is a change in the difference between theoretical and actual values, it can be inferred as a change in power loss.

Figure 3b presents these results. The black line represents the actual measured values, the blue line represents the theoretical values, and the red line shows the difference between them (ΔR). It can be observed that the difference in resistance increases as the via size decreases. As mentioned earlier, apart from the fixed resistance elements inherent in the measurement, additional resistance significantly increases as the via size decreases, indicating additional power loss. This means that reducing the via size results in additional power loss beyond the resistance determined by the material itself, and this lost power is expected to be dissipated as thermal energy.

## 4. Improvement of Model Structure and Simulation Methodology

In the previous section, we confirmed through resistance comparative analysis that as the area of the vias decreases, additional power losses occur, and the extent of these losses increases. These additional power losses are eventually converted into thermal energy and dissipated as heat [22]. We verified this through experimental validation and, using these results along with simulation analysis, we conducted further research to explore structures that minimize power loss within the specified metal line dimensions. This research was similarly verified through experiments.

We analyzed the improved structure samples designed through FEM analysis using the Ansys simulator. The analysis tools included electric and steady-state thermal analyses, each conducted as separate projects, with their results integrated to derive the data. When configuring the size of the simulation structure, we referred to the typical specifications of bumps and metal lines produced at the current process level. Bumps are usually manufactured according to micro bump standards, approximately 20 μm in size, while the metal lines directly connected to transistors have widths of around 2 nm. Therefore, between transistors and bumps, there are multiple layers of metal lines with widths ranging from 2 nm to 20 μm [23,24,25]. Among these, we adopted metal lines with a width of 15 μm as the standard for simulation specifications.

**Figure 4:**
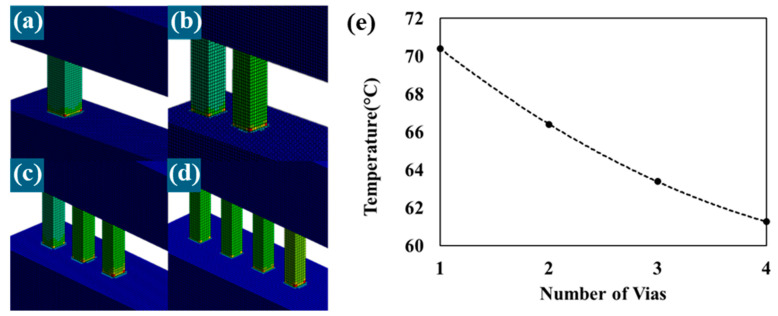
Improvement structure analyzed by FEM analysis through ANSYS simulation. The volume of vias was fixed and divided into several parts. (**a**) A general structure with one via with a width of 5 μm. (**b**) Two vias with a width of 3.54 μm. (**c**) Three vias with a width of 2.9 μm. (**d**) Four vias with a width of 2.5 μm. (**e**) Temperature reduction behavior profile by distribution of current density.

The connection between the metal lines eventually connects the upper and lower metals with a plug-like via. The most vulnerable point in the structure between metal lines and vias is the junction where they meet. Particularly in the junction of high-density current flow in metal lines and vias, the inner corner is the area most stressed by the flow of high-density current where EM primarily occurs. We concluded that optimizing the flow of high-density current to be properly distributed would alleviate stress at the junction. Therefore, in this study, we designed numerous samples with the shape and structure of the vias as variables and found the optimal improvement structure through FEM analysis. The adopted improvement structure is represented in Figure 4a–d. Under fixed conditions equal to the volume value of a typical via in Figure 4a, the number of vias increases parallel to the metal lines, as shown in Figure 4b–d. The adopted structure was expected to relax the temperature of the interconnecting structure as the current density was distributed in a cascading waterfall-like manner.

The Joule heat values obtained from electric analysis were utilized as simulation inputs in Ansys simulation, considering various material properties, to derive temperature results in steady-state thermal analysis. Upon closer examination of specific conditions, the simulation model incorporated Cu alloy properties (isotropic elasticity with a Young’s modulus of 1.1 × 10^5^ MPa, a Poisson ratio of 0.34, an isotropic thermal conductivity of 4.01 × 10^8^ pW/m·°C, and an isotropic electrical resistivity of 16.94 MW·m at 20 °C) provided by Ansys Simulation for the metal lines and vias. After selecting the materials, mesh conditions were configured for precise calculations. High-power components within the BEOL structure, our research target, experience elevated power densities leading to the generation of Joule heat. Consequently, the interconnects are exposed to extreme temperature changes, resulting in defects and reduced reliability. To accurately analyze the vulnerabilities of this BEOL structure and the temperature effects caused by Joule heating, we set up a mesh consisting of 220,000 cubic elements with a width of 0.5 μm across the entire sample model with 932,000 nodes. Each mesh and node element is individually calculated based on the applied currents and voltages and the set material properties to provide more accurate results. In this analysis based on approximately 220,000 meshes, the time required to extract the current density, Joule heating, and temperature changes in each mesh unit is about 5 min, allowing for the extraction of data through calculations at a rate of approximately 40,000 meshes per minute. It was confirmed that as the number of vias increased while maintaining a constant volume, the temperature due to Joule heating gradually decreased. In other words, the same magnitude of current applied to the entire simulation model was dispersed to each via, alleviating the current density and resulting in a decrease in the resistance generated at the junctions between the existing metal lines and vias. The data profile is shown in Figure 4e. The temperature of the data is the highest temperature generated by each model. As seen in Figure 4e, the peak resistance thermal temperature generated in the model with one via (Figure 4a) showed the largest decrease, decreasing by approximately 5 °C compared to the peak resistance thermal temperature in the model with two vias (Figure 4b), indicating the greatest reduction. The model with four vias (Figure 4d) exhibited the lowest peak temperature, but it is expected that further increases in the number of vias would lead to a gradual saturation of the temperature.

## 5. Result and Discussion

**Figure 5:**
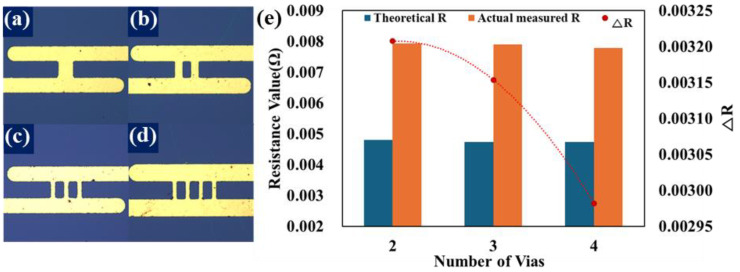
Models with increased number of vias implemented through the lift-off process: (**a**) Standard structure. (**b**–**d**) Improved structure models. (**e**) Resistance values and ΔR behavior profiles.

**Figure 6:**
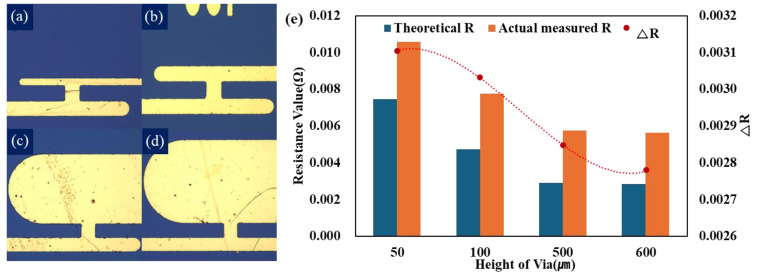
Models with increased upper metal line height implemented through the lift-off process: (**a**) Standard structure. (**b**–**d**) Improved structure. (**e**) Resistance values and ΔR behavior profiles.

With the aim of optimizing signal transmission, the improved structure was presented through simulation. It was verified power efficiency by applying the ΔR mechanism. Figure 5a–d and Figure 6a–d are optical microscope photographs of samples implemented by Cu deposition on a titanium layer in a TOP view. Figure 5a and Figure 6b are the general interconnect structure models to be compared in this experiment, and Figure 5b–d and Figure 6c,d are the improved structure models implemented based on FEM analysis in this experiment. The blue area is a wafer on which 300nm of SiO_2_ is deposited, and the yellow interconnect structure is a Cu interconnect. For the convenience of specimen manufacturing, the simulation model structure was constructed as a 2D structure rotated 90 degrees. The resulting data values of the 2D-structured model are the same as those extracted by making a 3D-structured model if a slight calculation is added. In other words, it has been implemented in a 2D structure that is more efficient to extract sufficient data with a 2D structure. In the resistance measurement model according to the change in the number of vias in Figure 5, the height of the upper and lower metal lines and the space between the vias were applied equally to all models under fixed conditions. The via line width of Figure 5a with a via line width of 100 μm is equal to the sum of the via line width of each model in Figure 5b–d.

The line width of vias was applied in a two-dimensional structure with increased process efficiency for the study, but assuming a three-dimensional structure, the sum of the volumes of each via of the models with two or more vias is equal to the volume of one via in the general structure. In Figure 6, in the resistance measurement model according to the height change in the upper metal line, only the height of the upper metal line was specified, and the size of the via and the height of the lower metal line were applied under fixed conditions. The upper metal line model was also implemented in a 2D structure, but like the previous via improvement model, it was applied as a volume change in 3D. In this experiment, the following models were implemented, and theoretical R and actual measured R were extracted using calculation and probe station equipment, respectively. The degree of improvement in the current transfer of each model was evaluated by applying the ΔR mechanism, which verified the improvement in current transfer with the tendency of ΔR extracted through calculation.

Figure 5e is the data profile of the via improvement structure applying the ΔR mechanism. The measured resistance of the models with two to four vias was found to be about twice as low as that of the model with 1 via. The difference in resistance between via improved structural models is found to be fine, but both improved structures tend to have lower theoretical resistance than the actual measured resistance. The ΔR of the via improved structural model showed a tendency to decrease as the number of vias increased, as we expected. These results can generally be said to have improved power efficiency. Resistance is one of the factors that directly affect power loss. The lower the resistance, the less heat loss occurs during current flow [26]. Power loss occurs due to a variety of factors, one of which is when electrical energy is converted into heat. In other words, the higher the resistance, the faster the electrical energy is converted into heat, and the higher the resistance, the higher the heat loss. On the other hand, as the rate of heat loss is slowed, the temperature due to the resistance heat decreases and the power loss decreases. This means that the current density generated when the current flows through the interconnect improvement structure in Figure 5b–d is relaxed compared to the general structure, and the resistance is measured low, resulting in a decrease in the temperature caused by Joule heating.

We anticipated that an increase in the height of the upper metal line receiving the first power transfer would reduce resistance due to the relaxation of current density, and at the same time, the flow of current to via, which decreases sharply compared to the width of the wider metal line, would result in high resistance. In the previous experiments, we observed the effect of vias, but we also wanted to verify the impact of reducing the current density applied to the vias as a method to improve power efficiency, even when the vias cannot be modified. Basically, the metal line of the BEOL’s interconnect structure, which connects the I/O terminal to the transistor, has a shape in which the scaling decreases as it approaches the transistor, and there are metal lines of various heights. Therefore, we additionally measured the resistance according to the height change in this upper metal line. The resistance measurement model, according to the height change in the upper metal line, also selected the same general interconnect structure model (Figure 6b) as in Figure 5a for the resistance measurement model, using the change in the number of vias as a comparison target. Figure 6e is the data profile of the metal line’s improvement structure applying the ΔR mechanism.

The overall resistance data trend of the resistance measurement models according to the height change in the upper metal line indicates that the theoretical resistance tends to be higher as opposed to the resistance measurement model resistance data according to the number of vias in the front. This trend can be used as the width of resistance due to process variables between model making. A model with a height of 50 μm (Figure 6a) was built for comparison with Figure 6c,d with a height of 500 μm and 600 μm. Looking at the behavior of ΔR in Figure 6e, the overall trend is decreasing, and ΔR is quadratic in form between the 50 μm and 100 μm models and between the 500 μm and 600 μm models. The highest resistance was extracted from the model in Figure 6a, which is 50 μm high, and the resistance generated decreases as the height increases gradually in the order of 100 μm (Figure 6b), 500 μm (Figure 6c), and 600 μm (Figure 6d). The largest resistance was shown between the 50 μm height model and the 100 μm height model, and there was a slight difference between the 500 μm and 600 μm height models. In addition, looking at the behavior of ΔR, it was confirmed that the graph trend was reversed between the 100 μm height model and the 500 μm height model, and the behavior of ΔR was saturated between the 500 μm and 600 μm height models. This means that an increase in the height of the metal line above a certain height does not have a significant effect. In summary, the increase in the height of the upper metal line influences the resistance reduction, but it is judged that the width of the resistance reduction becomes saturated when the height increases above a certain height.

The resistance measurement models according to the change in the number of vias and the height of the upper metal line generally showed a tendency to decrease ΔR as the number of vias increased and the height of the upper metal line increased. These trends indicate the opposite behavior of the decrease in ΔR data of the model due to the increase in AR between the metal line of the general structure and the via. When evaluated from the perspective of the ΔR mechanism, the upper metal line height increase model and the number of vias evaluated in this study generally show lower resistance than the general structure. It can be said that the current transfer has been optimized. In addition, it was proved by matching the temperature trend of Joule heating in the simulation conducted earlier.

## 6. Conclusions

This paper proposes a method for analyzing the signal transmission efficiency in interconnect structures and, based on this, presents improved structures to optimize signal transmission efficiency. The simulation results predict and confirm that increasing the number of vias or the thickness of the upper metal lines in the interconnects reduces power loss when the same number of current flows. As semiconductor systems become more highly integrated, the width of the interconnects decreases, and their complexity increases, highlighting the growing importance of optimizing interconnects. The interconnect and junction structures that make up semiconductor systems ultimately have a relatively simple form, consisting of the bonding between metal layers [27,28,29,30,31]. The findings of this study provide insights into improving power efficiency in the connection between two metal layers and the plug that connects them, making it practical for real-world applications. Based on the results of this study, we have identified ways to improve the signal transmission efficiency in interconnects by altering their structure without the need for extreme process development, even when the same current flows through them.

These findings are expected to contribute significantly to identifying easier improvement measures through a deeper understanding of the components that constitute semiconductor systems.

## Data Availability

The raw data supporting the conclusions of this article will be made available by the authors on request.
